# Effectiveness of a Web-Based Educational eHealth Platform on Women’s Health Literacy About Phthalate Exposure: Randomized Controlled Trial

**DOI:** 10.2196/87063

**Published:** 2026-09-01

**Authors:** Ya Ling Shih, Shu Ching Wu, Pei Shan Li, Chia Jung Hsieh, Chieh-Yu Liu

**Affiliations:** 1 School of Nursing, College of Medicine and Nursing, Yuan Ze University, Taoyuan, Taiwan; 2 Department of Nursing, Cheng Hsin General Hospital, Taipei, Taiwan; 3 Department of Long-Term Care, College of Health Technology, National Taipei University of Nursing and Health Sciences, Taipei, Taiwan; 4 School of Nursing, College of Nursing, National Taipei University of Nursing and Health Sciences, No. 365, Mingde Road, Beitou District, Taipei, 112303, Taiwan, 886 02-2822-7101 ext 3109; 5 Institute of Community Health Care, National Yang Ming Chiao Tung University, Taipei, Taiwan

**Keywords:** eHealth, health literacy, phthalates, women’s health, randomized controlled trial

## Abstract

**Background:**

Phthalates are environmental endocrine-disrupting chemicals widely used in plastics, cosmetics, food packaging, and personal care products. Women may experience frequent exposure through everyday consumer and household products. Improving phthalate-related health literacy may support informed exposure-reduction decisions; however, conventional health education provides limited opportunities for repeated, interactive, and individually tailored learning.

**Objective:**

This randomized controlled trial evaluated the effectiveness of an eHealth educational intervention (Phthalates Free) in improving women’s overall and domain-specific phthalate-related health literacy and examined the association between platform engagement and health literacy outcomes.

**Methods:**

A double-blind randomized controlled trial was conducted in the outpatient department of a regional teaching hospital in Taipei, Taiwan. A total of 114 women were randomly assigned to an intervention group (n=58) receiving a 6-month eHealth platform–based education program and a control group (n=56) receiving conventional paper-based education. Assessments were conducted at baseline (T0), 3 months (T1), and 6 months (T2). The Phthalate Health Literacy Scale (10 items; *α*=.90, content validity index=0.93) measured overall and domain-specific literacy (health care, disease prevention, and health promotion). Longitudinal outcomes were analyzed using generalized estimating equations based on all available observations according to participants’ original randomized assignments, with adjustment for waist circumference and pregnancy history. Analysis of covariance (ANCOVA) was used to compare 6-month outcomes after adjustment for baseline scores. Platform engagement and perceived usability were assessed using back-end analytics and the System Usability Scale (SUS).

**Results:**

At 6 months, the intervention group showed a significantly greater increase in total health literacy than the control group (+9.93 points, Wald *χ*²_1_=17.74; *P*<.001). Domain analyses revealed significant improvements in health care (+1.52; *P*=.001), disease prevention (+1.32; *P*=.001), and health promotion (+1.12; *P*=.001) domains. ANCOVA confirmed the between-group difference at T2 after adjusting for baseline scores (*F*_1,109_=11.43; *P*=.001; adjusted mean difference=7.15, 95% CI 2.96‐11.34). Engagement analysis showed that high-engagement users (n=10) scored significantly higher in overall health literacy (*t*_55_=–3.00; *P*=.004) and all domains than general users. The SUS results (mean 84.7, SD 5.2; n=46, 79.3%) indicated high perceived usability.

**Conclusions:**

The Phthalates Free eHealth educational intervention significantly improved women’s overall and domain-specific health literacy over 6 months. Higher platform engagement was associated with better health literacy outcomes. The intervention may serve as a practical adjunct to nurse-led education in outpatient and community settings by providing accessible, continuous, and evidence-based guidance on reducing phthalate exposure.

## Introduction

Phthalates, a ubiquitous class of environmental hormones, are widely used in daily consumer products such as plastics, cosmetics, food packaging, and medical devices. Extensive epidemiological and toxicological research has shown that phthalate exposure is associated with endocrine disruption, reproductive abnormalities, metabolic syndrome, and neurodevelopmental disorders [1-16]. Owing to their concealed presence and cumulative toxicity, public awareness regarding phthalate sources, health effects, and preventive measures remains limited. Women are particularly vulnerable because of their frequent use of cosmetics and personal care products, and this knowledge gap may further compromise their ability to manage personal and family health [4,16,17].

Health literacy has been identified by the World Health Organization as a key determinant of health, encompassing individuals’ ability to access, understand, appraise, and apply health information [18,19]. Improved environmental hormone-related health literacy has been shown to reduce high-risk behaviors, encourage healthier dietary practices, and foster positive lifestyle changes [20]. However, conventional approaches, such as printed materials and one-time face-to-face education, may provide limited opportunities for repeated, interactive, and individually tailored learning compared with eHealth interventions [21].

The rapid advancement of digital technology and mobile health tools has expanded opportunities for innovative health education. eHealth platforms have been widely applied to programs for chronic disease management, health promotion, and environmental health literacy, providing scalability, interactivity, and traceability [22-25].

Previous interventions have addressed phthalate and endocrine-disrupting chemical exposure through behavioral guidance and digital education. Chen et al [26] reported that practical behavioral strategies among Taiwanese girls were associated with reductions in urinary phthalate metabolites, whereas Kim et al [27] found that a web-based intervention for mothers with young children reduced several biomarkers of endocrine-disrupting chemical exposure. Tomsho et al [28] demonstrated that a virtual educational intervention improved phthalate-related environmental health literacy and patient counseling among reproductive health professionals. In addition, a scoping review by Yang et al [29] identified a range of interventions targeting phthalates, synthetic phenols, and related chemicals and demonstrated that exposure reduction may be achievable through educational, dietary, product-replacement, and behavioral strategies.

Although these studies provide important evidence supporting environmental exposure education, several gaps remain. Previous interventions have generally focused on selected exposure-reduction behaviors, short-term biomarker changes, mothers with young children, children, or health professionals. Relatively limited evidence is available regarding theory-based, longitudinal eHealth interventions designed specifically to strengthen adult women’s multidimensional phthalate-related health literacy. In particular, few studies have comprehensively addressed the abilities to access, understand, appraise, and apply phthalate-related information across the health care, disease prevention, and health promotion domains or examined whether improvements are sustained over time.

To address this gap, we used a user-centered approach to develop Phthalates Free, an eHealth educational platform designed to improve women’s environmental health literacy regarding phthalate exposure. The platform integrates structured nursing education, multimedia learning materials, interactive quizzes, feedback, reminders, and practical behavioral guidance to provide accessible and actionable exposure-reduction strategies. This randomized controlled trial (RCT) evaluated the effectiveness of the intervention in improving women’s overall and domain-specific phthalate-related health literacy over 6 months. We hypothesized that women receiving the eHealth intervention would demonstrate greater improvements in phthalate-related health literacy than those receiving conventional paper-based education.

## Methods

### Study Design

This study was designed as an interventional RCT with a double-blind design, in which both participants and the principal investigator or statistical analyst were unaware of group assignments. The trial aimed to compare the effectiveness of an electronic nursing guidance platform on environmental hormones—specifically phthalates (eHealth intervention)—with that of conventional paper-based health education in improving women’s phthalate-related health literacy. The intervention period lasted 6 months, with assessments conducted at baseline (T0), 3 months after the intervention (T1), and 6 months after the intervention (T2). The study was reported in accordance with the CONSORT-EHEALTH (Consolidated Standards of Reporting Trials of Electronic and Mobile Health Applications and Online Telehealth) checklist (Checklist 1).

### Sample Size Estimation

The required sample size was estimated using G*Power (version 3.0) for a repeated-measures design (*F* test, effect size *f*=0.25, 2-tailed *α*=.05, power=0.80, 3 measurement points). The minimum required sample size was 86 participants in total. Considering an estimated 30% attrition rate due to the COVID-19 pandemic, the target sample size was set at 120 participants (60 per group).

### Participants and Setting

Participants were recruited from the outpatient departments of obstetrics and gynecology and family medicine at a regional teaching hospital in Taipei City, Taiwan. After obtaining institutional approval and ethics clearance, trained research assistants recruited participants on-site using convenience sampling. A total of 120 women were assessed for eligibility; 6 were excluded, and 114 eligible participants were randomized.

Inclusion criteria were as follows: (1) women aged 20 years or older, (2) ability to communicate in Mandarin or Taiwanese, and (3) willingness to participate with signed informed consent. Exclusion criteria included being male, being younger than 20 years of age, or having a diagnosed psychiatric or cognitive disorder. Eligible participants were randomly assigned to either the intervention group (n=58) or the control group (n=56).

### Randomization and Blinding

A sequentially numbered, opaque, sealed envelope procedure was used for randomization and allocation concealment. The random allocation sequence and envelopes were prepared and safeguarded by an independent research assistant who was not involved in participant recruitment, outcome assessment, data management, or statistical analysis. After eligibility was confirmed and the baseline assessment was completed, envelopes were opened sequentially according to enrollment order.

Participants were informed only that they would receive health education and follow-up related to environmental hormones and phthalate exposure. They were not informed that the study included 2 different intervention conditions and were not told of their formal group allocation. Although participants were aware of the educational materials they personally received, they did not know whether those materials corresponded to the intervention or control condition.

Separate research assistants were assigned exclusively to the intervention and control groups and were responsible for intervention delivery. The principal investigator did not participate in sequence generation, envelope opening, participant allocation, or intervention delivery. Group identities were masked using coded labels in the analytic dataset, and the allocation codes were disclosed to the principal investigator and the statistical analyst only after the completion of the primary analyses.

### Intervention and Control

#### Intervention Group: eHealth Nursing Guidance Platform

**Figure 1.**
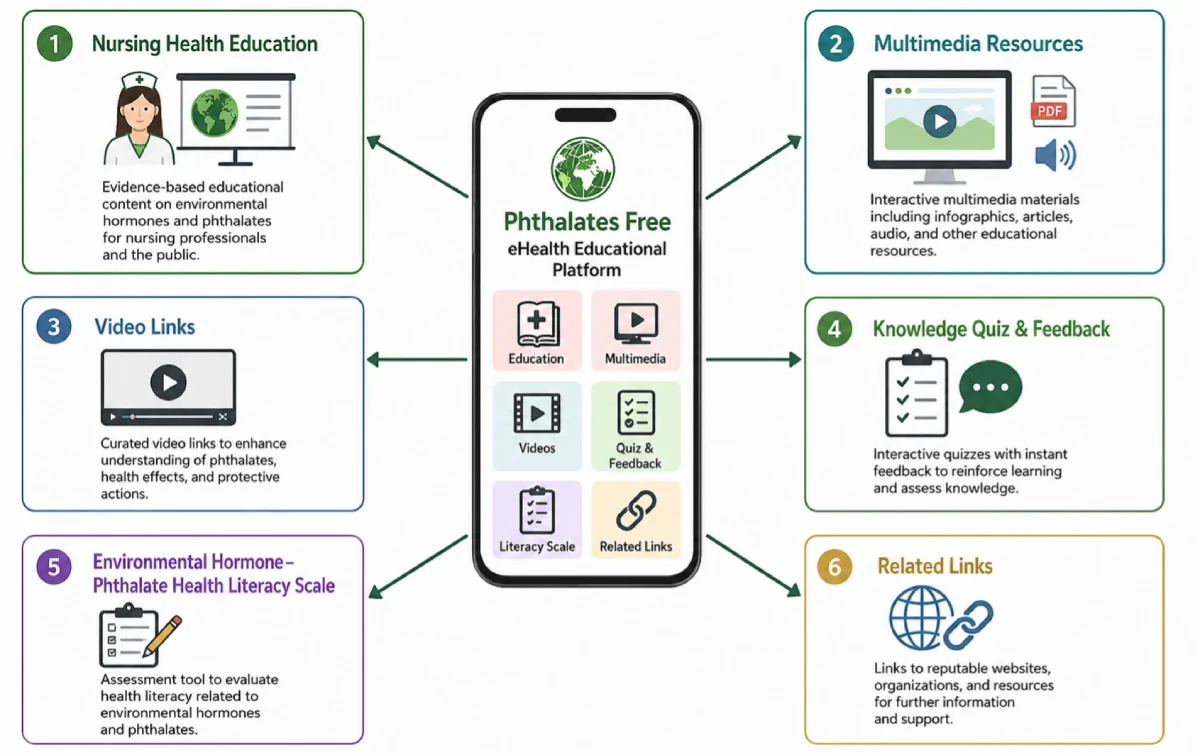
Components of the Phthalates Free eHealth educational platform.

Participants in the intervention group received access to a user-centered eHealth nursing guidance platform on environmental hormones and phthalates. The platform was hosted on Google Workspace, incorporating cloud infrastructure with identity and access management, single sign-on, and data loss prevention protocols. It was integrated with an official LINE account (“Environmental Hormones—Phthalates Free”) to enable personalized communication and engagement. The intervention components are shown in Figure 1, with detailed screenshots provided in Multimedia Appendix 1.

The platform design followed the World Health Organization’s definition of eHealth, emphasizing the application of information and communication technologies for health promotion and disease management [30], and was guided by the 9 design dimensions proposed by Dijkman et al [31], including target-group orientation, evidence-based content, interactivity, and privacy protection.

#### Platform Functional Modules

The eHealth nursing guidance platform consisted of five functional modules designed to support knowledge acquisition, engagement, and application of phthalate-related health information.

1. Educational module: Provided evidence-based information on definitions, sources, uses, exposure routes, health risks, and avoidance strategies [32].
2. Lifestyle guidance: The knowledge-behavior gap refers to the discrepancy between knowing about the health risks of phthalate exposure and consistently translating that knowledge into exposure-reduction behaviors in daily life. This gap may arise because phthalates are embedded in routine activities and commonly used products and because individuals may not know which feasible alternatives to adopt. To address this gap, the platform translated general risk information into context-specific and actionable guidance for diet, personal care, clothing, home environments, and cleaning practices. Examples included avoiding heating food in plastic containers, selecting glass or stainless-steel food containers, checking personal care product labels, reducing the use of fragranced products, improving household ventilation, and using damp cleaning methods to reduce contaminated dust [20,23].
3. Multimedia learning: Short videos, infographics, and linked resources were provided with the intention of supporting learning motivation and comprehension, based on evidence regarding multimedia health education [21,33].
4. Interactive features: Push notifications, segmented communication, Q&A responses, and self-administered questionnaires were incorporated to support participant engagement [22,34].
5. Usage analytics: Back-end logging of login frequency, duration, and feature clicks provided quantitative indicators of engagement for subsequent analysis [35].

Participants received structured nursing guidance through the platform, including remote consultations via LINE, telephone, or email. A 30-minute baseline counseling session was conducted before intervention initiation. During the 6-month period, participants received periodic push notifications and personalized feedback. Outcome assessments were performed at T1 and T2 during clinic visits or online sessions.

This eHealth design was grounded in empirical evidence supporting the effectiveness of digital health interventions in enhancing chronic disease management and health promotion outcomes [36,37]. These design features were adapted for environmental health education and were intended to support women’s phthalate-related health literacy and daily exposure-reduction decision-making.

#### Theoretical Framework

This study was grounded in the Integrated Model of Health Literacy, which delineates 4 core competencies—access, understand, appraise, and apply—that enable individuals to make informed health decisions and maintain well-being [18]. The eHealth platform was conceptually aligned with these competencies through multimedia learning materials, interactive assessments, LINE-based consultations, and real-time feedback mechanisms.

Specifically, the platform operationalized the Integrated Model of Health Literacy through four digital design components—each corresponding to a key literacy competency:

1. Access: The platform provided timely, evidence-based health information through visually engaging multimedia formats, including text, infographics, and short videos.
2. Understand: Educational content was presented using concise language, structured headings, infographics, illustrations, and short videos to support comprehension and reduce cognitive burden. The understandability and actionability of the slide presentations and educational videos were retrospectively evaluated using the Patient Education Materials Assessment Tool for Printable Materials (PEMAT-P) [38] and Patient Education Materials Assessment Tool for Audiovisual Materials (PEMAT-A/V) [39], respectively.
3. Appraise: Interactive quizzes and tailored push notifications encouraged users to critically reflect on their health behaviors and evaluate the credibility and relevance of the information received.
4. Apply: Contextualized case scenarios and practical lifestyle reminders were designed to support the application of phthalate-related information in daily exposure-reduction decision-making.

By integrating structured education, multimedia resources, feedback, reminders, and practical guidance, the platform was designed to support users’ access to, understanding of, appraisal of, and application of phthalate-related health information. The lifestyle guidance component was intended to help users translate risk information into feasible daily exposure-reduction practices.

#### Control Group: Usual Care

Participants in the control group received standard paper-based health education and routine verbal counseling consistent with clinical practice. They did not have access to the eHealth platform or interactive digital features. Both groups completed identical assessments at T1 and T2 during outpatient visits or online follow-ups to ensure comparability.

#### Assessment of Educational Material Understandability and Actionability

The understandability and actionability of the educational materials were retrospectively assessed using the Patient Education Materials Assessment Tool (PEMAT). A total of 11 nursing education slide presentations were evaluated using the PEMAT-P [38], whereas 4 educational videos were evaluated using the PEMAT-A/V [39]. The PEMAT evaluates whether patient education materials are understandable to individuals with varying levels of health literacy and whether the materials clearly identify actions that users can take. Each applicable item was rated as agree or disagree, and items considered not applicable were excluded from the denominator. Separate percentage scores for understandability and actionability were calculated for each educational material.

Two reviewers independently evaluated each material. One reviewer had expertise in nursing informatics and digital health education, whereas the other had expertise in public health and health education. Disagreements were resolved through discussion and consensus. Mean scores and ranges were subsequently calculated separately for the slide presentations and educational videos.

### Outcome Measures

#### Baseline Health Assessment

At baseline, participants completed a structured questionnaire that collected demographic, physiological, and lifestyle information. Demographic variables included age, education level, marital status, and occupation. Physiological indicators comprised height, weight, blood pressure, pulse rate, waist circumference, and BMI. Lifestyle variables covered exercise habits, alcohol consumption, smoking status, sleep duration, water intake, and bowel habits, along with family and personal medical history, reproductive history, and medication use.

#### Primary Outcome: Phthalate Health Literacy Scale

The primary outcome was measured using the Phthalate Health Literacy Scale, consisting of 10 items derived from the conceptual framework of health literacy [18] and designed to assess phthalate-related health literacy in culturally and contextually relevant situations in Taiwan. These situations included potential exposure through plastic food containers, polyvinyl chloride products, food packaging, cosmetics, personal care products, and household products. The scale assessed 3 domains—health care, disease prevention, and health promotion—using a 4-point Likert-type scale ranging from 1 (“very difficult”) to 4 (“very easy”). Scores were converted to a standardized range of 0 to 100, with higher scores indicating greater health literacy.

The scale demonstrated good psychometric properties: content validity index=0.93; Cronbach *α*=0.90 (subscales 0.73‐0.79); test-retest reliability=0.80 (*P*<.001); confirmatory factor analysis fit indices comparative fit index=0.97, normed fit index=0.94, goodness-of-fit index=0.88; criterion validity with the 12-item Short-Form Health Literacy Questionnaire (HL-SF12; *r*=0.59; *P*<.001). Known-group validity further demonstrated significant differences according to educational level, supporting the scale’s ability to identify variations in health literacy among Taiwanese women [40].

All questionnaires were administered electronically through a secure online survey interface. Responses were automatically uploaded to and stored in a secure Google Workspace database, which reduced manual data-entry errors and supported data integrity.

#### Secondary Outcomes

##### Engagement

Platform usage was tracked via back-end analytics (login frequency, feature clicks, duration, and response activity). Participants were expected to log in weekly during month 1 and twice monthly from months 2 to 6 (14 times in total) and were categorized as general or highly engaged for outcome analysis.

##### System Usability

At T2, participants in the intervention group completed the 10-item System Usability Scale (SUS), originally developed by Brooke [41]. Each item used a 5-point Likert-type scale, with total scores converted to 0 to 100, where higher scores reflected better usability.

##### Data Collection and Follow-Up Procedures

Data were collected at 3 time points: T0 (baseline), T1 (3 mo), and T2 (6 mo). These intervals were selected to assess both the early and sustained effects of the intervention. The baseline assessment was conducted before the assigned health education was initiated. The 3-month assessment was used to identify early changes after repeated exposure to the educational content, whereas the 6-month assessment corresponded to the completion of the full intervention period and was used to determine whether improvements in phthalate-related health literacy were sustained over time. At T0, participants completed baseline assessments; the intervention group additionally received platform orientation and their first counseling session. At T1 and T2, both groups completed the Phthalate Health Literacy Scale; the intervention group also completed the SUS at T2. Participants were contacted via telephone, email, or LINE for reminders and follow-up. All interaction data were automatically logged.

##### Data Management and Quality Assurance

All survey data were coded and anonymized before analysis. Two independent research assistants performed double data entry and cross-verification, and any discrepancies were resolved by reviewing the original questionnaires. Platform usage data were securely managed within the Google Workspace environment and protected through identity and access management, single sign-on, and data loss prevention protocols to restrict unauthorized access and data sharing. The research team conducted regular data quality checks to identify missing or abnormal values. Any inconsistencies detected were verified against the original data sources to ensure accuracy and completeness of the dataset. All digital and physical data were stored in encrypted, access-controlled environments. Personal identifiers were removed prior to analysis, and only authorized investigators could access the deidentified dataset. Confidentiality was maintained throughout data collection, storage, and publication according to institutional review board (IRB) and national data-protection regulations.

### Statistical Analysis

Analyses were conducted in SPSS (version 26.0; IBM Corp). Continuous data were expressed as means (SD) and categorical data as frequencies (%). Baseline differences were assessed using 2-tailed *t* tests and *χ*² tests. Within-group changes (T0-T1-T2) were analyzed via paired 2-tailed *t* tests, and between-group differences via independent 2-tailed *t* tests. Longitudinal effects were examined using generalized estimating equations (GEE) including group, time, and group × time interaction, controlling for significant baseline covariates. Analysis of covariance (ANCOVA) was additionally performed to compare total and domain-specific health literacy scores between groups at 6 months after adjustment for the corresponding baseline scores. The homogeneity-of-regression-slopes assumption was examined before each ANCOVA. Adjusted mean differences and 95% CIs were reported. Participants were analyzed according to their original randomized group assignments. GEE analyses incorporated all available observations from the 3 assessment points without imputing missing outcome data. Participant attrition was reported at each time point. All statistical tests were 2-tailed, with a significance level of *α*=.05.

### Ethical Considerations

This study was approved by the Institutional Review Board of Cheng Hsin General Hospital, Taipei, Taiwan (CHGH-IRB number (864)110-10) and conducted in accordance with the Declaration of Helsinki. All participants provided written informed consent prior to enrollment and could withdraw at any time without affecting their medical rights. The data were anonymized and securely stored with access limited to authorized researchers. The trial was registered at ClinicalTrials.gov (NCT05024227).

### Reporting Standards

This manuscript was prepared in accordance with the CONSORT-EHEALTH extension [42] to ensure comprehensive and transparent reporting of this web-based RCT.

## Results

### Participant Characteristics

**Figure 2.**
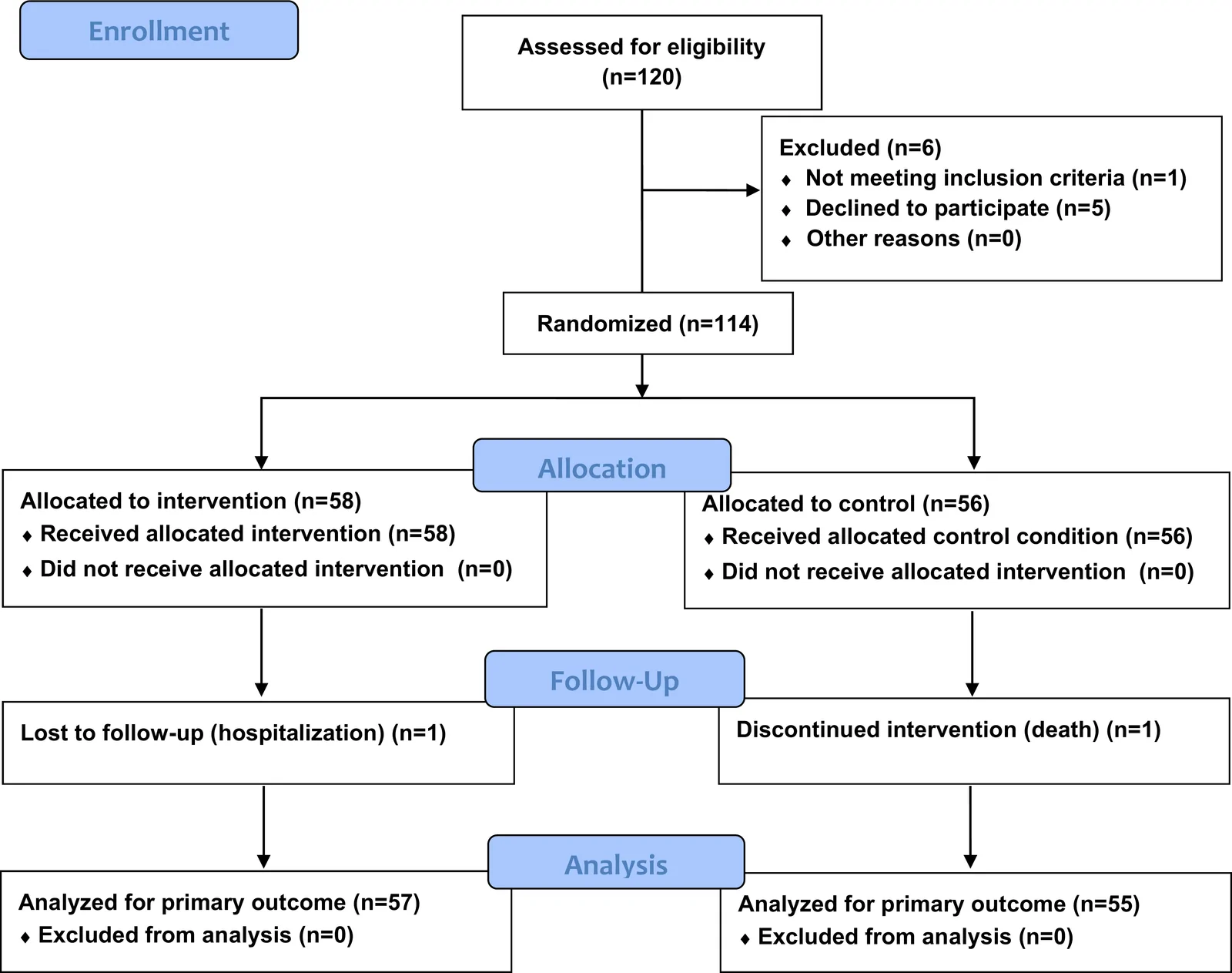
CONSORT (Consolidated Standards of Reporting Trials) flow diagram of participant enrollment, allocation, follow-up, and analysis.

A total of 114 women were enrolled and randomized in the intervention group (n=58) and the control group (n=56). After the 6-month follow-up, 1 participant from each group withdrew, resulting in 112 participants who completed the study (intervention group, n=57; control group, n=55; Figure 2).

At baseline, there were no significant differences between groups in most demographic or physiological characteristics, indicating good group homogeneity. The mean age of participants was 36.9 (SD 12.3) years (intervention group: 35.0, SD 10.7; control group: 38.8, SD 13.7; *P*=.098). The mean BMI was 23.3 (SD 4.5) in the intervention group and 24.7 (SD 5.3) in the control group (*P*=.12). Overall, 59.60% (n=68) of the participants were single, and 91.20% (n=104) had a college-level education or higher. The most frequently reported family medical histories were metabolic syndrome (n=77, 67.50%) and diabetes (n=57, 50.00%; Table 1).

**Table 1.** Baseline characteristics and homogeneity test of the two groups.

Variable	All (n=114)	EG^a^ (n=58)	CG^b^ (n=56)	*t* test (*df*)	*P* value
Age, mean (SD)	36.88 (12.31)	35 (10.65)	38.82 (13.65)	−1.67 (112)	.098
Height, mean (SD)	159.74 (5.16)	159.94 (5.49)	159.54 (4.84)	0.41 (112)	.68
Weight, mean (SD)	61.5 (13.69)	59.88 (13.41)	63.17 (13.89)	−1.29 (112)	.20
Systolic blood pressure, mean (SD)	118.54 (12.33)	116.23 (13.01)	120.8 (13.39)	−1.63 (112)	.11
Diastolic blood pressure, mean (SD)	71.46 (11.21)	69.34 (10.73)	73.53 (11.40)	−1.79 (112)	.08
Pulse, mean (SD)	77.67 (10.26)	76.83 (9.31)	78.53 (11.19)	−0.79 (112)	.43
Waist circumference (cm), mean (SD)	77.71 (14.36)	73.89 (9.31)	80.51 (15.71)	−2.11 (112)	.04^c^
Average daily working hours, mean (SD)	8.41 (2.57)	8.65 (2.35)	8.17 (2.78)	0.99 (112)	.32
Sleep duration (h), mean (SD)	6.79 (1.10)	6.94 (1.08)	6.63 (1.11)	1.53 (112)	.13
Daily water intake (mL), mean (SD)	1468.14 (752.26)	1425 (667.76)	1513.64 (836.02)	−0.62 (112)	.53
Menarche, mean (SD)	12.97 (1.52)	13.05 (1.52)	12.89 (1.53)	0.66 (112)	.53
Menopause, mean (SD)	50.14 (5.76)	51.33 (4.46)	49.25 (6.74)	0.56 (15)	.58
BMI, mean (SD)	23.98 (4.93)	23.27 (4.48)	24.71 (5.30)	−1.57 (112)	.12
Marital status, n (%)				—^d^	.09
Single	68 (59.60)	39 (67.20)	29 (51.80)		
Nonsingle	46 (40.60)	19 (32.80)	27 (48.20)		
BMI, n (%)				—	.45
<18.5	10 (8.80)	7 (12.10)	3 (5.40)		
18.5≤ BMI <24	57 (50.00)	28 (48.30)	29 (51.80)		
≥24	47 (41.20)	23 (39.60)	24 (42.80)		
Education, n (%)				—	.09
Elementary + middle school	4 (3.50)	0 (0.00)	4 (7.10)		
High school	6 (5.30)	2 (3.40)	4 (7.10)		
College	87 (76.30)	43 (74.10)	44 (78.60)		
Graduate school or over	17 (14.90)	13 (22.40)	4 (7.10)		
Medical history, n (%)				—	.47
No	69 (60.50)	37 (63.80)	32 (57.10)		
Yes	45 (39.50)	21 (36.20)	24 (42.90)		
Family history, n (%)				—	.77
No	9 (7.90)	5 (8.60)	4 (7.10)		
Yes	105 (92.10)	53 (91.40)	52 (92.90)		
Metabolic syndrome, n (%)	77 (67.50)	41 (70.70)	36 (64.30)	—	.47
Diabetes, n (%)	57 (50.00)	30 (51.70)	27 (48.20)	—	.71
Parkinson disease, n (%)	4 (3.50)	3 (5.20)	1 (1.80)	—	.33
Heart disease, n (%)	21 (18.40)	14 (24.10)	7 (12.50)	—	.11
Stroke, n (%)	20 (17.50)	8 (13.80)	12 (21.40)	—	.28
Kidney disease, n (%)	4 (3.50)	3 (5.20)	1 (1.80)	—	.33
Cancer, n (%)	38 (33.30)	20 (34.50)	18 (32.10)	—	.79
Autoimmune disease, n (%)	6 (5.30)	2 (3.40)	4 (7.10)	—	.38
Gastrointestinal disease, n (%)	7 (6.10)	2 (3.40)	5 (8.90)	—	.22
Symptoms in the past 6 months, n (%)					
No	13 (11.40)	6 (10.30)	7 (12.50)	—	.72
Pulmonary disease	27 (23.70)	16 (27.60)	11 (19.60)	—	.73
Cardiovascular disease	64 (56.10)	37 (63.80)	27 (48.20)	—	.51
Gastrointestinal disorder	38 (33.40)	18 (30.90)	20 (35.80)	—	.25
Shoulder or neck pain or fatigue	68 (59.60)	39 (67.20)	29 (51.80)	—	.09
Irregular menstruation	30 (26.30)	11 (19.00)	19 (33.90)	—	.07
Occupation, n (%)				—	.16
Military, public service, or teaching	10 (8.80)	2 (3.40)	8 (14.30)		
Service industry	32 (28.10)	19 (32.80)	13 (23.20)		
Business, wholesale, or retail	7 (6.10)	2 (3.40)	5 (8.90)		
Technology R&D	2 (1.80)	1 (1.70)	1 (1.80)		
Health care	42 (36.80)	26 (44.80)	16 (28.60)		
Student or housewife	10 (8.80)	5 (8.60)	5 (8.90)		
Retired or unemployed	4 (3.50)	1 (1.70)	3 (5.40)		
Others	7 (6.10)	2 (3.40)	5 (8.90)		
Workplace, n (%)				—	.14
Indoor	78 (68.40)	42 (72.40)	36 (64.30)		
Outdoor	8 (7.00)	1 (1.70)	7 (12.50)		
Factory	1 (0.90)	0 (0.00)	1 (1.80)		
Hospital	26 (22.80)	14 (24.10)	12 (21.40)		
Laboratory	1 (0.90)	1 (1.70)	0 (0.00)		
Fatigue status, n (%)				—	.06
Never	3 (2.60)	2 (3.40)	1 (1.80)		
Rarely	27 (23.70)	7 (12.10)	20 (35.70)		
Sometimes	51 (44.70)	29 (50.00)	22 (39.30)		
Often	23 (20.20)	14 (24.10)	9 (16.10)		
Always	10 (8.80)	6 (10.30)	4 (7.10)		
Bowel habits, n (%)				—	.28
Once/day	62 (54.40)	32 (55.20)	30 (53.60)		
Twice/day	17 (14.90)	5 (8.60)	12 (21.40)		
More than twice/day	1 (0.90)	1 (1.70)	0 (0.00)		
Once every 2 days	23 (20.20)	14 (24.10)	9 (16.10)		
Less than once every 2 days	11 (9.60)	6 (10.40)	5 (8.90)		
Exercise habits, n (%)				—	.25
No	81 (71.10)	44 (75.90)	37 (66.10)		
Yes	33 (28.90)	14 (24.10)	19 (33.90)		
Smoking habits, n (%)				—	.08
No or occasionally	109 (95.60)	53 (91.40)	56 (100.00)		
Quit	2 (1.80)	2 (3.40)	0 (0.00)		
Yes	3 (2.60)	3 (5.20)	0 (0.00)		
Drinking habits, n (%)				—	.31
None or occasionally	113 (99.10)	58 (100.00)	55 (98.20)		
Currently ongoing	1 (0.90)	0 (0.00)	1 (1.80)		
Weight control, n (%)				—	.95
Reduction	33 (28.90)	17 (29.30)	16 (28.60)		
Maintenance	52 (45.60)	27 (46.60)	25 (44.60)		
No	29 (25.40)	14 (24.10)	15 (26.80)		
Dietary supplement use, n (%)				—	.13
Never	24 (21.10)	10 (17.20)	14 (25.00)		
Rarely	31 (27.10)	15 (25.90)	16 (28.60)		
Sometimes	16 (14.00)	8 (13.80)	8 (14.30)		
Often	21 (18.40)	16 (27.60)	5 (8.90)		
Always	24 (21.10)	10 (17.20)	14 (25.00)		
Health-seeking behavior, n (%)				—	.46
Medical institutions	28 (24.60)	13 (22.40)	15 (26.80)		
Use of over-the-counter medicine	8 (7.00)	5 (8.60)	3 (5.40)		
No medication use	2 (1.80)	2 (3.40)	0 (0.00)		
Depending on the situation	76 (66.70)	38 (65.50)	38 (67.00)		

a EG: experimental group.

b CG: control group.

c *P*<.05.

d Not applicable.

However, there were significant differences between groups in the waist circumference (intervention group: 73.9, SD 9.3 cm; control group: 80.5, SD 15.7 cm; *P*=.038) and pregnancy experience (n=22, 37.90% vs n=36, 64.30%; *P*=.005; Table 2). These variables were therefore included as covariates in subsequent analyses to control for potential confounding effects.

Overall, both groups were comparable in baseline demographics, lifestyle behaviors, disease history, and reproductive characteristics, supporting the validity of the subsequent intervention outcome comparisons.

**Table 2.** Homogeneity analysis of female-related characteristics between the two groups.

Variable	All, n (%)	EG^a^, n (%)	CG^b^, n (%)	*P* value
Menstrual cycle				.46
Regular	31 (27.20)	14 (24.10)	17 (30.40)	
Irregular	83 (72.80)	44 (75.90)	39 (69.60)	
Current menstruation				.73
No	17 (14.90)	8 (13.80)	9 (16.10)	
Yes	97 (85.10)	50 (86.20)	47 (83.90)	
Pregnancy experience				.005^c^
No	56 (49.10)	36 (62.10)	20 (35.70)	
Yes	58 (50.90)	22 (37.90)	36 (64.30)	
Use of hormonal medications				.84
No	99 (86.80)	50 (86.20)	49 (87.50)	
Yes	15 (13.20)	8 (13.80)	7 (12.50)	
Use of Chinese herbal medicines for women’s health				.07
No	82 (71.90)	46 (79.30)	36 (64.30)	
Yes	32 (28.10)	12 (20.70)	20 (35.70)	
Use of estrogen, placenta extract, evening primrose oil, or related supplements				.22
No	107 (93.90)	56 (96.60)	51 (91.10)	
Yes	7 (6.10)	2 (3.40)	5 (8.90)	
Gynecological conditions				.62
No	81 (71.10)	40 (69.00)	41 (73.20)	
Yes	33 (28.90)	18 (31.00)	15 (26.80)	
Severe dysmenorrhea	7 (6.10)	5 (8.60)	2 (3.60)	.26
Uterine fibroids	10 (8.80)	5 (8.60)	5 (8.90)	.95
Ovarian cysts	5 (4.40)	1 (1.70)	4 (7.10)	.16
Endometriosis (chocolate cyst or adenomyosis)	11 (9.60)	7 (12.10)	4 (7.10)	.37
Uterine or cervical polyps	3 (2.60)	0 (0.00)	3 (5.40)	.07

a EG: experimental group.

b CG: control group.

c *P*<.05.

### Effects of the eHealth Intervention on Health Literacy

**Figure 3.**
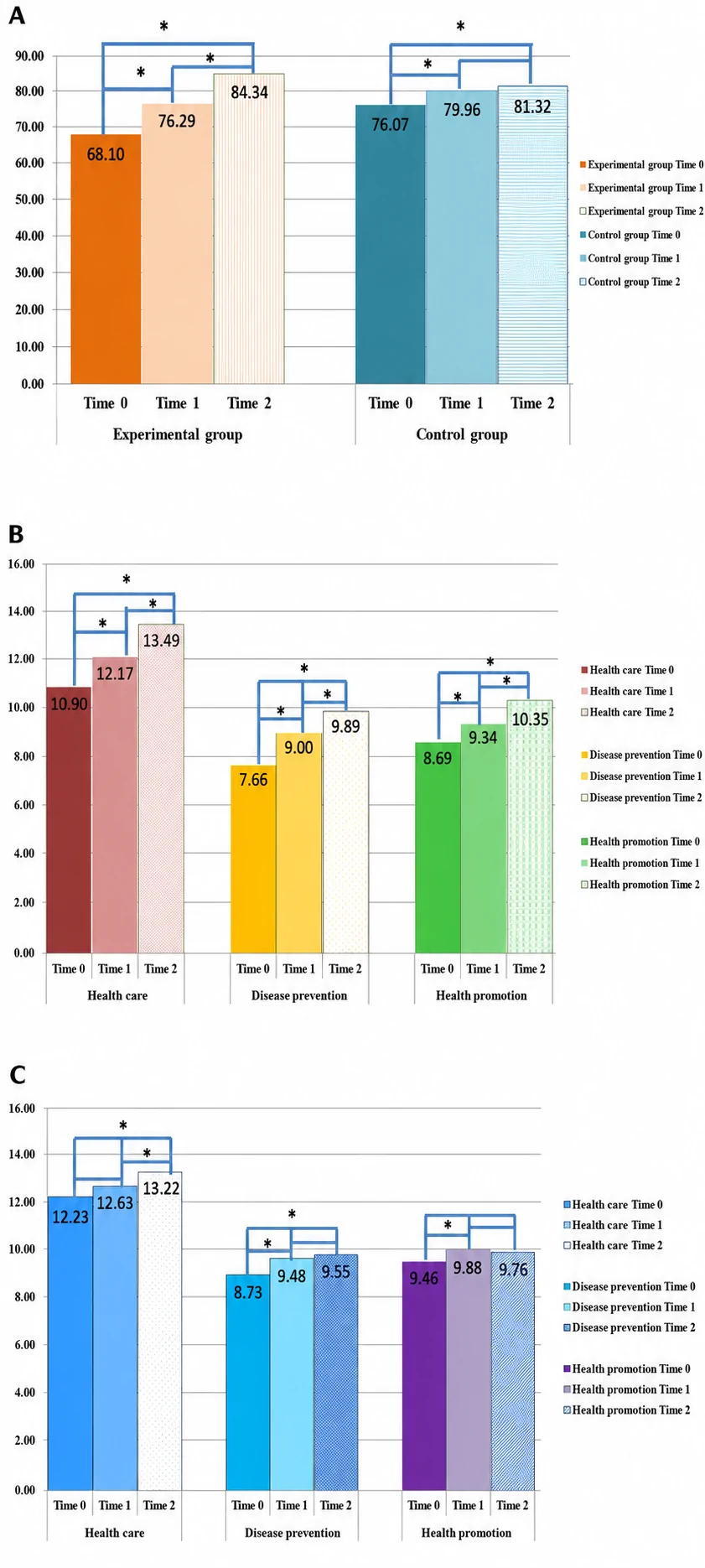
Overall health literacy and domain-specific comparisons between the groups. (A) Overall health literacy trends in the experimental and control groups. (B) Domain-specific health literacy trends in the experimental group. (C) Domain-specific health literacy trends in the control group. *P<.05.

Phthalate-related health literacy was assessed at baseline (T0), 3 months (T1), and 6 months (T2). The intervention group’s mean total health literacy score increased from 68.10 (SD 9.76) at T0 to 76.29 (SD 7.77) at T1 and 84.34 (SD 10.76) at T2, whereas the control group’s score increased from 76.07 (SD 13.73) to 79.96 (SD 13.97) and 81.32 (SD 12.51), respectively. Although the intervention group had a lower baseline score, the difference between the groups decreased over time. At T2, the intervention group also had a significantly higher unadjusted score in the health promotion domain than the control group (10.35 vs 9.76; *P*=.04; Figure 3).

### GEE and ANCOVA Results

The adjusted GEE analysis showed a significant group × time interaction for total health literacy at T2. Compared with the control group, the intervention group demonstrated an additional increase of 9.93 points from T0 to T2 (95% CI 5.31‐14.56; Wald *χ*²_1_=17.74; *P*<.001). Significant group × time interactions were also observed in the health care (*β*=1.52, 95% CI 0.65‐2.38; *P*=.001), disease prevention (*β*=1.32, 95% CI 0.57‐2.07; *P*=.001), and health promotion domains (*β*=1.12, 95% CI 0.43‐1.80; *P*=.001; Table 3).

**Table 3.** Generalized estimating equation (GEE) analysis of health literacy score changes between groups.

Variable	Estimate, ß (SE; 95% CI)	Wald χ² (*df*)	*P* value
Overall health literacy			
Intercept	42.57 (13.64; 15.83 to 69.30)	9.74 (1)	.002^a^
Time			
Posttest (T2) vs pretest (T0)	6.18 (1.96; 2.35 to 10.01)	9.98 (1)	.002^a^
Mid-test (T1) vs pretest (T0)	5.49 (1.6; 2.36 to 8.62)	11.82 (1)	.001^a^
Group			
EG^b^ vs CG^c^	−8.73 (2.44; −13.51 to −3.94)	12.79 (1)	<.001^d^
Interaction (group × time)			
Group × time (posttest [T2] vs pretest [T0])^e^	9.93 (2.36; 5.31 to 14.56)	17.74 (1)	<.001^d^
Group × time (mid-test [T1] vs pretest [T0])^f^	2.57 (1.94; −1.23 to 6.36)	1.76 (1)	.19
Health care domain			
Intercept	7.87 (2.73; 2.52 to 13.22)	8.31 (1)	.004^a^
Time			
Posttest (T2) vs pretest (T0)	1.15 (0.36; 0.45 to 1.85)	10.36 (1)	.001^a^
Mid-test (T1) vs pretest (T0)	0.54 (0.33; −0.1 to 1.18)	2.77 (1)	.096
Group			
EG vs CG	−1.58 (0.43; −2.43 to −0.73)	13.24 (1)	<.001^d^
Interaction (group × time)			
Group × time (posttest [T2] vs pretest [T0])^e^	1.52 (0.44; 0.65 to 2.38)	11.77 (1)	.001^a^
Group × time (mid-test [T1] vs pretest [T0])^f^	0.75 (0.4; −0.03 to 1.52)	3.53 (1)	.06
Disease prevention domain			
Intercept	4.26 (1.73; 0.86 to 7.65)	6.04 (1)	.01^g^
Time			
Posttest (T2) vs pretest (T0)	0.93 (0.31; 0.32 to 1.54)	8.90 (1)	.003^a^
Mid-test (T1) vs pretest (T0)	1.02 (0.25; 0.54 to 1.50)	17.40 (1)	<.001^d^
Group			
EG vs CG	−1.12 (0.36; −1.82 to −0.41)	9.69 (1)	.002^a^
Interaction (group × time)			
Group × time (posttest [T2] vs pretest [T0])^e^	1.32 (0.38; 0.57 to 2.07)	11.86 (1)	.001^a^
Group × time (mid-test [T1] vs pretest [T0])^f^	0.29 (0.32; −0.34 to 0.92)	0.81 (1)	.37
Health promotion domain			
Intercept	5.01 (1.45; 2.17 to 7.85)	11.97 (1)	.001^a^
Time			
Posttest (T2) vs pretest (T0)	0.42 (0.28; −0.12 to 0.96)	2.29 (1)	.13
Mid-test (T1) vs pretest (T0)	0.63 (0.23; 0.19 to 1.08)	7.74 (1)	.005^a^
Group			
EG vs CG	−0.77 (0.34; −1.44 to −0.09)	4.96 (1)	.03^g^
Interaction (group × time)			
Group × time (posttest [T2] vs pretest [T0])^e^	1.12 (0.35; 0.43 to 1.80)	10.15 (1)	.001^a^
Group × time (mid-test [T1] vs pretest [T0])^f^	−0.01 (0.31; −0.61 to 0.60)	0 (1)	.98

a *P*<.01.

b EG: experimental group.

c CG: control group.

d *P*<.001.

e {EG (posttest [T2] vs pretest [T0])} vs {CG (posttest [T2] vs pretest [T0])}.

f {EG (mid-test [T1] vs pretest [T0])} vs {CG (mid-test [T1] vs pretest (T0])}.

g *P*<.05.

**Figure 4.**
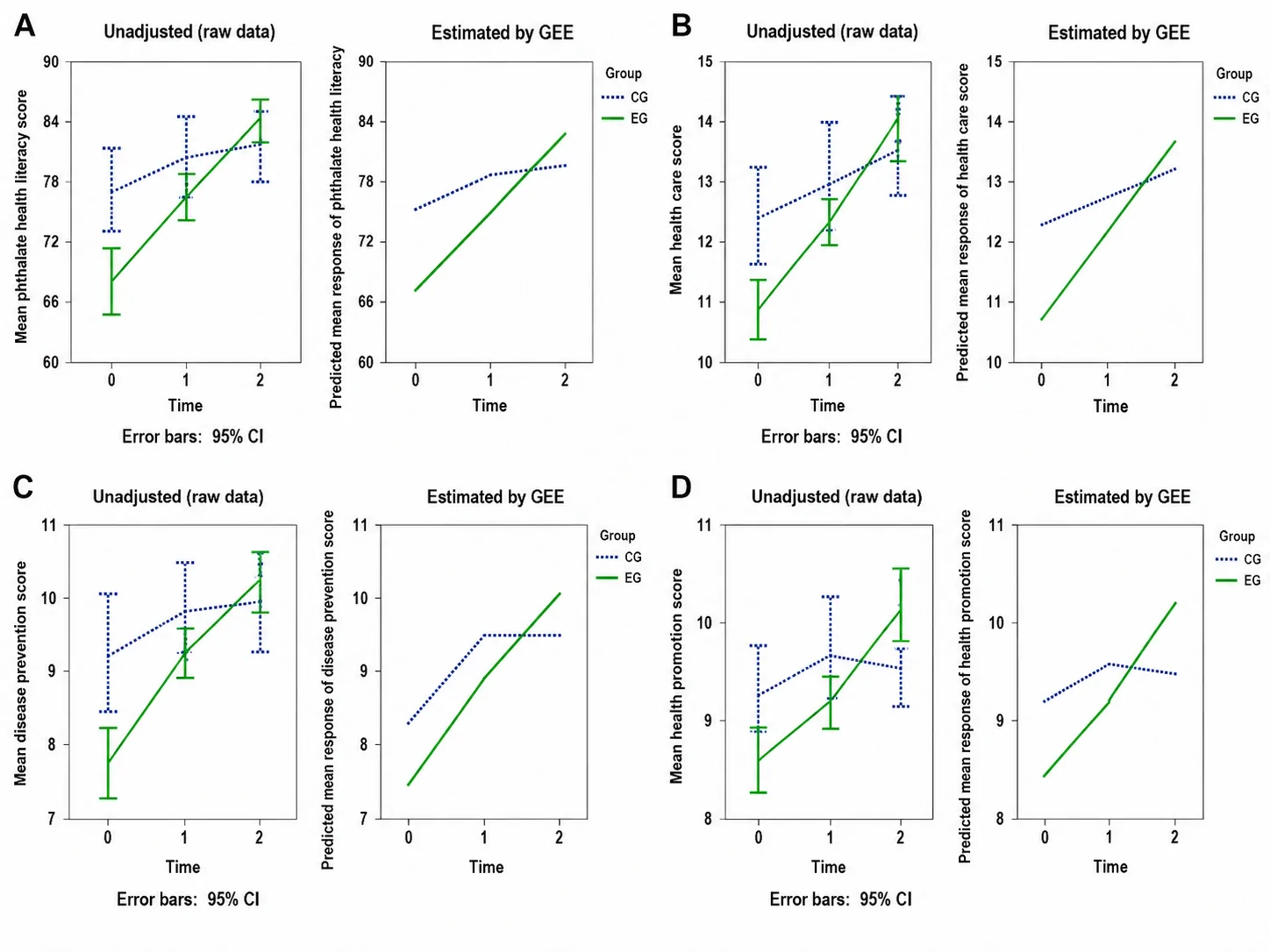
Unadjusted and generalized estimating equation (GEE)–estimated changes in overall and domain-specific phthalate-related health literacy over time. (A) Overall health literacy, (B) health care domain, (C) disease prevention domain, (D) health promotion domain. The left panels show unadjusted group means with SD error bars at baseline (T0), 3 months (T1), and 6 months (T2). The right panels show GEE-estimated trajectories for the experimental group (EG) and control (CG) group.

ANCOVA results were consistent with the GEE findings. After adjustment for baseline scores, the intervention group had significantly higher total health literacy at T2 than the control group (*F*_1,109_=11.43; *P*=.001; adjusted mean difference 7.15, 95% CI 2.96‐11.34). Significant adjusted between-group differences were also observed in the health care (adjusted mean difference 0.74, 95% CI 0.04‐1.43; *P*=.04), disease prevention (0.88, 95% CI 0.23‐1.52; *P*=.008), and health promotion domains (0.90, 95% CI 0.37‐1.44; *P*=.001; Table 4). Changes in total and domain-specific phthalate-related health literacy over time in the intervention and control groups are illustrated in Figure 4.

**Table 4.** Analysis of covariance (ANCOVA) of overall health literacy at 6 months postintervention.

Variable	Sum of squares	Mean square	*F* (*df*)	*P* value
Overall health literacy				
Pretest	3097.68	3097.68	28.54^a^ (1,109)	>.001
Group	1240.29	1240.29	11.43^b^ (1,109)	.001^b^
Error	11,832.08	108.55	—^c^	—
Adjusted total	15,185.71	—	—	—
Health care domain				
Pretest	44.48	44.48	14.64 (1,109)	<.001
Group	13.37	13.37	4.40 (1,109)	.04^d^
Error	331.15	3.04	—	—
Adjusted total	377.71	—	—	—
Disease prevention domain				
Pretest	59.78	59.78	22.69 (1,109)	<.001
Group	19.07	19.07	7.24 (1,109)	.008^b^
Error	287.22	2.64	—	—
Adjusted total	350.42	—	—	—
Health promotion domain				
Pretest	40.60	40.60	21.04 (1,109)	<.001
Group	21.41	21.41	11.09 (1,109)	.001^b^
Error	210.31	1.93	—	—
Adjusted total	260.56	—	—	—

a *P*<.001.

b *P*<.01.

c Not applicable.

d *P*<.05.

### Secondary Outcomes

#### Engagement and Phthalate-Related Health Literacy

At T2, participants with high platform engagement (n=10) had significantly higher scores than those with general engagement (n=47) in total health literacy (93.00 vs 82.50; *P*=.004), health care (14.90 vs 13.19; *P*=.003), disease prevention (10.90 vs 9.68; *P*=.026), and health promotion (11.40 vs 10.13; *P*=.009; Table 5).

**Table 5.** Differences in health literacy and domain scores by participation level (experimental group).

Outcome and time point	General participation (n=47), mean (SD)	High participation (n=10), mean (SD)	*t* test (*df*)	*P* value
Overall health literacy				
Time 0	67.45 (9.93)	71.25 (8.68)	−1.12 (55)	.27
Time 1	75.83 (7.50)	78.50 (9.07)	−0.99 (55)	.33
Time 2	82.50 (9.84)	93.00 (11.17)	−3.00 (55)	.004^a^
Health care domain				
Time 0	10.81 (1.70)	11.30 (1.57)	−0.84 (55)	.41
Time 1	12.19 (1.18)	12.10 (1.85)	0.14 (55)	.89
Time 2	13.19 (1.57)	14.90 (1.60)	−3.12 (55)	.003^a^
Disease prevention domain				
Time 0	7.56 (1.51)	8.10 (1.37)	−1.04 (55)	.31
Time 1	8.90 (1.22)	9.50 (1.72)	−1.32 (55)	.19
Time 2	9.68 (1.49)	10.90 (1.73)	−2.29 (55)	.03^b^
Health promotion domain				
Time 0	8.60 (1.61)	9.10 (1.20)	−0.92 (55)	.36
Time 1	9.25 (1.12)	9.80 (1.14)	−1.41 (55)	.16
Time 2	10.13 (1.35)	11.40 (1.35)	−2.72 (55)	.009^a^

a *P*<.01.

b *P*<.05.

#### Understandability and Actionability of the Educational Materials

The 11 nursing education slide presentations achieved a mean PEMAT-P understandability score of 78.4% (SD 6.8; range 67.7%-91.2%) and a mean actionability score of 71.4% (SD 28.9; range 28.6%-100.0%). The 4 educational videos achieved a mean PEMAT-A/V understandability score of 86.5% (SD 3.9; range 84.6%-92.3%) and a mean actionability score of 68.8% (SD 12.5; range 50.0%‐75.0%).

#### System Usability Assessment

At T2, 46 of the 58 (79.30%) participants in the intervention group completed the SUS. The mean SUS score was 84.7 (SD 11.0), indicating usability well above the commonly cited average benchmark of 68 [43]. Because 68 represents an approximate average rather than a formal pass-fail threshold, this score indicates above-average perceived usability but does not imply that the platform was universally usable across all users or settings.

## Discussion

This section interprets the study findings within theoretical, empirical, and practical contexts. It discusses the mechanisms underlying intervention effectiveness; compares results with prior and recent evidence; and outlines implications for clinical practice, policy translation, and future research in digital health education and environmental health literacy.

### Principal Findings

This randomized, double-blind controlled trial provides empirical evidence that an eHealth educational intervention can effectively enhance women’s environmental health literacy related to phthalate exposure, with significant improvements across all 3 literacy domains—health care, disease prevention, and health promotion.

A key finding was the positive association between user engagement and learning outcomes. Participants with higher platform engagement had higher total and domain-specific health literacy scores at T2. This finding suggests that user engagement may be an important correlate of learning reinforcement and health literacy outcomes. Frequent exposure to interactive educational content may facilitate habit formation, enhance cognitive retention, and strengthen motivation to adopt preventive behaviors—critical processes in the knowledge-to-action continuum.

These results suggest that digital health platforms, when designed according to theoretical models such as the Integrated Model of Health Literacy, can successfully operationalize the 4 competencies of accessing, understanding, appraising, and applying health information and were associated with measurable improvements in phthalate-related health literacy.

### Comparison With Prior Work

Our findings are consistent with the broader literature indicating that digital health interventions can improve health literacy by supporting users’ ability to access, understand, appraise, and apply health information. Previous studies have shown that web- and app-based educational programs incorporating multimedia content, interactivity, and repeated access can strengthen health literacy, self-management, motivation, and confidence across diverse populations [20,23-25,44].

However, the present study addresses a distinct and comparatively understudied area: health literacy related to environmental exposure. Phthalate exposure remains an important environmental health concern because of its associations with endocrine disruption, metabolic disorders, reproductive abnormalities, and hormone-sensitive cancers [5,45,46]. Although Taiwan strengthened regulatory controls following the 2011 plasticizer contamination incident, biomonitoring studies have continued to document phthalate exposure among Taiwanese adults and pregnant women [4,17,47-49].

Women may experience additional exposure through frequent contact with cosmetics, personal care products, and household materials. Therefore, an eHealth intervention specifically designed to strengthen women’s ability to identify, interpret, evaluate, and apply phthalate-related health information is both timely and relevant. The integration of structured educational content, behavioral guidance, multimedia materials, quizzes, and repeated digital engagement is consistent with established principles of technology-assisted health education. Nevertheless, because evidence from general digital health literacy interventions does not directly demonstrate effectiveness for environmental exposure reduction, the findings of the present study should be interpreted primarily in relation to prior interventions addressing phthalates and other endocrine-disrupting chemicals.

### Comparison With Environmental Exposure Education Interventions

Previous interventions addressing phthalates and other endocrine-disrupting chemicals have used educational and behavioral strategies to improve exposure-related knowledge and reduce potentially harmful practices. In Taiwan, Chen et al [26] developed 7 behavioral strategies for girls with elevated phthalate exposure, including more frequent handwashing, avoiding plastic containers and plastic food coverings, refraining from microwaving food in plastic, and reducing the use of cosmetics and personal care products. Following the intervention, several exposure-reduction behaviors were associated with lower urinary concentrations of specific phthalate metabolites. These findings indicate that concrete and context-specific behavioral guidance may contribute to measurable reductions in exposure.

Digital approaches have further expanded the delivery of environmental exposure education. Kim et al [27] conducted an RCT of a web-based behavioral intervention among mothers with young children. The intervention included educational videos, an interactive game for identifying endocrine disruptors in the home, information on potential exposure sources, online resources, and a question-and-answer component. Compared with written information, the web-based program resulted in reductions in several urinary biomarkers, including phthalate metabolites, bisphenol A, and parabens after 1 month. This study supports the value of combining environmental health information with interactive tools and practical exposure-reduction guidance.

Internet-based environmental education has also been applied during pregnancy. In a nonrandomized controlled study, Kim et al [50] delivered an environmental prenatal education program through videoconferencing. The program improved perceived environmental severity, response efficacy, and overall environmental health perception, although significant changes in personal and community environmental behaviors were not observed. These findings suggest that improvements in awareness and risk perception may precede behavioral change and that sustained reinforcement may be required to translate knowledge into routine preventive practices.

More recently, Tomsho et al [28] developed a 1-hour virtual educational intervention for reproductive health professionals covering routes of phthalate exposure, associated reproductive health effects, and strategies for discussing exposure reduction with patients. The intervention improved phthalate-related environmental reproductive health literacy and substantially increased the reported frequency with which clinicians discussed phthalates with patients. This work illustrates how virtual education can strengthen not only individual knowledge but also the communication of environmental health information within clinical practice.

Collectively, these studies are consistent with our findings that technology-assisted environmental health education can improve the capacity to understand and respond to environmental exposure risks. However, our study extends the existing evidence in several ways. First, the intervention was specifically structured around the Integrated Model of Health Literacy, addressing the competencies of accessing, understanding, appraising, and applying phthalate-related information. Second, the platform combined structured nursing education, audiovisual materials, quizzes, reminders, and practical lifestyle guidance over a 6-month period rather than providing a single brief educational session. Third, the sustained improvement observed across the health care, disease prevention, and health promotion domains suggests that repeated digital engagement may support the maintenance of environmental health literacy beyond immediate postintervention effects.

Nevertheless, evidence across environmental exposure interventions remains heterogeneous. A scoping review by Yang et al [29] identified 26 interventions targeting synthetic phenols, glycol ethers, and phthalates, but only 6 relied solely on education; many studies instead removed or replaced exposure-related products. This distinction is important because educational interventions depend on participants’ ability and opportunity to translate information into behavioral change, whereas product-replacement interventions directly alter exposure sources. Our findings therefore contribute evidence that a scalable educational approach can produce sustained improvements in phthalate-related health literacy, although future studies should determine whether these improvements also lead to objectively measured reductions in exposure.

### Behavioral Mechanisms and Practical and Policy Implications for Digital Health Education and Promotion

The positive association between platform engagement and literacy outcomes observed in this study suggests that user engagement may contribute to the effectiveness of digital health interventions. Repeated interactions with multimedia learning materials, quizzes, and tailored push notifications likely reinforced cognitive retention, strengthened motivation, and cultivated self-regulatory behaviors. These iterative feedback loops fostered reflective learning and enabled participants to internalize information, potentially supporting the application of phthalate-related information in daily decision-making. Such findings align with the knowledge-to-action continuum described in the Integrated Model of Health Literacy, wherein sustained exposure, self-efficacy, and reflection collectively facilitate durable behavioral change [18,50].

Nursing interventions, through structured education and health promotion strategies, have long been recognized as essential in addressing low health literacy and improving behavioral outcomes [51]. Within this framework, eHealth platforms represent an evolution of traditional nursing education, extending the reach of health professionals beyond physical clinical boundaries. By integrating behavioral feedback and evidence-based guidance, these platforms facilitate accessible, personalized, and continuous learning experiences. The Phthalates Free platform, for example, incorporated real-time feedback, tailored reminders, and interactive media that allowed participants to engage repeatedly and autonomously—features particularly conducive to adult learning and habit formation. No technical failures, data breaches, or operational disruptions occurred during the trial, further confirming the platform’s stability, usability, and data security safeguards.

From a clinical perspective, this model promotes patient empowerment by integrating information access with active engagement. Nurses and health educators can leverage such tools to reinforce counseling, enhance autonomy, and provide ongoing communication that strengthens preventive behaviors. Continuous reinforcement through digital channels—particularly those embedded within daily communication platforms such as LINE—bridges the gap between episodic clinical encounters and long-term self-management. This aligns with contemporary nursing practice emphasizing participatory care, patient engagement, and evidence-informed prevention [52].

At the public health and policy level, the findings underscore the potential of digital health interventions as scalable, cost-effective tools for promoting environmental health literacy. The Phthalates Free model can inform the development of national or regional digital campaigns targeting women and other vulnerable populations, particularly where traditional outreach channels are limited. Leveraging existing digital infrastructures—such as social media, mobile messaging systems, and eHealth portals—facilitates the broad dissemination of validated health information with minimal resource investment. Furthermore, embedding digital literacy competencies within health promotion policies directly supports several Sustainable Development Goals (SDGs), including SDG 3 (Good Health and Well-being), SDG 5 (Gender Equality), and SDG 12 (Responsible Consumption and Production).

Finally, behavioral insights derived from platform analytics—such as engagement frequency, completion rates, and interaction patterns—offer a powerful foundation for adaptive policy design. Tailoring public health messages based on real-time behavioral data can enhance adherence, minimize information fatigue, and improve message salience among target populations. Integrating behavioral analytics with population-level surveillance systems may further enable policymakers to identify knowledge gaps, optimize the timing and delivery of interventions, and design adaptive communication strategies that maximize public engagement. Collectively, these findings highlight how digitally enabled nursing and public health education can function as a sustainable bridge connecting evidence generation, individual empowerment, and population-level policy translation.

### Generalizability

The findings of this RCT may be generalizable to other populations with comparable digital access and educational backgrounds. As the intervention was delivered through a widely used communication platform (LINE) and designed for mobile accessibility, it is applicable to general internet users and community-based health education settings. However, the generalizability to populations with limited internet access, lower digital literacy, or different cultural contexts may be restricted.

It should also be noted that several features implemented in the trial—such as automated push notifications, in-app reminders, and limited human assistance from research staff—were designed to enhance user engagement within the controlled setting. In routine applications without these supportive components, engagement levels and intervention effectiveness might be reduced. Future real-world deployment should therefore consider adaptive mechanisms (eg, AI-driven reminders or community health worker follow-ups) to maintain motivation and ensure sustainable outcomes across diverse populations.

### Strengths and Limitations

This study’s strengths include its randomized, double-blind controlled design, which minimized bias and enhanced causal inference. The use of both GEE and ANCOVA models strengthened analytical robustness by accounting for within-subject correlations and baseline differences. Furthermore, the integration of engagement metrics provided valuable insights into digital behavior and learning mechanisms, allowing a nuanced understanding of how participation influences outcomes.

However, several limitations should be acknowledged. The study was conducted in a single hospital setting in northern Taiwan, which may limit generalizability to other populations or cultural contexts. Although the Phthalate Health Literacy Scale was specifically developed and validated among Taiwanese women, further psychometric evaluation is warranted before applying it to populations with different cultural, linguistic, educational, or demographic characteristics. Health literacy outcomes were based on self-reported measures, potentially introducing social desirability bias. Due to time and funding constraints, biological indicators (eg, urinary phthalate metabolites) were not collected, preventing direct validation of whether improved literacy led to reduced exposure. The 6-month follow-up period captures short-term effects but may not reflect long-term sustainability.

Finally, although a double-blind protocol and procedural separation were implemented, differences in the educational delivery format may have influenced participant engagement. The Hawthorne effect also cannot be entirely excluded, as awareness of study participation and follow-up may have affected participants’ responses.

### Future Research Directions

Future research should aim to replicate these findings across multicenter, multiethnic, and cross-cultural settings to enhance external validity. Extending the intervention period to 12 months or longer would allow examination of the maintenance and decay patterns of health literacy and behavior change over time. Incorporating objective biomarkers, such as urinary or serum phthalate metabolite levels, would further establish the link between literacy improvement and biological outcomes.

As digital health technology evolves, future interventions should consider incorporating gamification, AI-driven personalization, and social network features to strengthen sustained engagement. Integration with wearable devices or mobile sensors could enable real-time exposure tracking and personalized feedback, connecting environmental data with health behavior analytics.

At the educational level, integrating digital health literacy modules into nursing curricula would equip health care professionals with the competencies needed to design, implement, and evaluate technology-assisted interventions. Cross-sector collaborations among health care, public health, engineering, and data science professionals are recommended to develop sustainable and adaptive eHealth ecosystems.

### Conclusions

This RCT demonstrated that the Phthalates Free eHealth intervention improved women’s phthalate-related health literacy over 6 months across the health care, disease prevention, and health promotion domains. Greater platform engagement was associated with better literacy outcomes. The intervention may serve as a practical adjunct to nurse-led education in outpatient and community settings by providing accessible, continuous, and evidence-based guidance on reducing phthalate exposure. Further multicenter studies involving populations with more diverse educational and digital literacy backgrounds are needed to confirm its broader applicability.

## Supplementary material

10.2196/87063 Multimedia Appendix 1 Screenshots of the Phthalates Free eHealth educational platform.

10.2196/87063 Checklist 1 Completed CONSORT‐EHEALTH (V1.6.1) checklist.

## Abbreviations

ANCOVA: analysis of covariance
CONSORT-EHEALTH: Consolidated Standards of Reporting Trials of Electronic and Mobile Health Applications and Online Telehealth
GEE: generalized estimating equation
HL-SF12: 12-item Short-Form Health Literacy Questionnaire
IRB: institutional review board
PEMAT: Patient Education Materials Assessment Tool
PEMAT-A/V: Patient Education Materials Assessment Tool for Audiovisual Materials
PEMAT-P: Patient Education Materials Assessment Tool for Printable Materials
PVC: polyvinyl chloride
RCT: randomized controlled trial
SDG: Sustainable Development Goal
SUS: System Usability Scale
