# Supplementary material for: Effectiveness of a Web-Based Educational eHealth Platform on Women’s Health Literacy About Phthalate Exposure: Randomized Controlled Trial
Source: J Med Internet Res. 2026 Sep 1;28:e87063. doi: 10.2196/87063 (PMC13533579; doi:10.2196/87063)
Supplement: Supplementary file 1 [file jmir-v28-e87063-s001.pdf]

Multimedia Appendix 1. Screenshots of the Phthalates Free eHealth Educational Platform

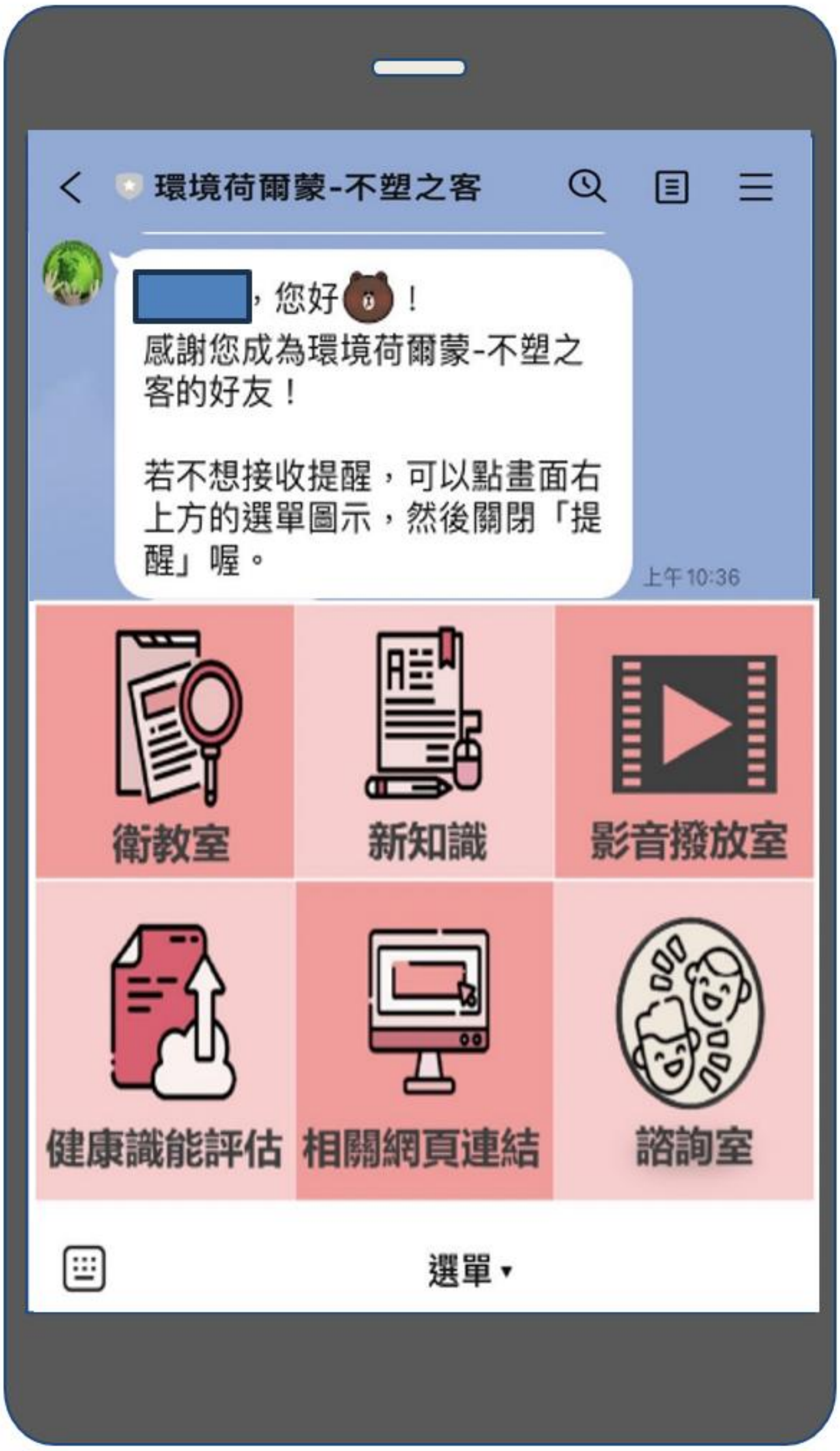

S1. Platform home page and navigation interface

環境荷爾蒙-塑化劑小教室

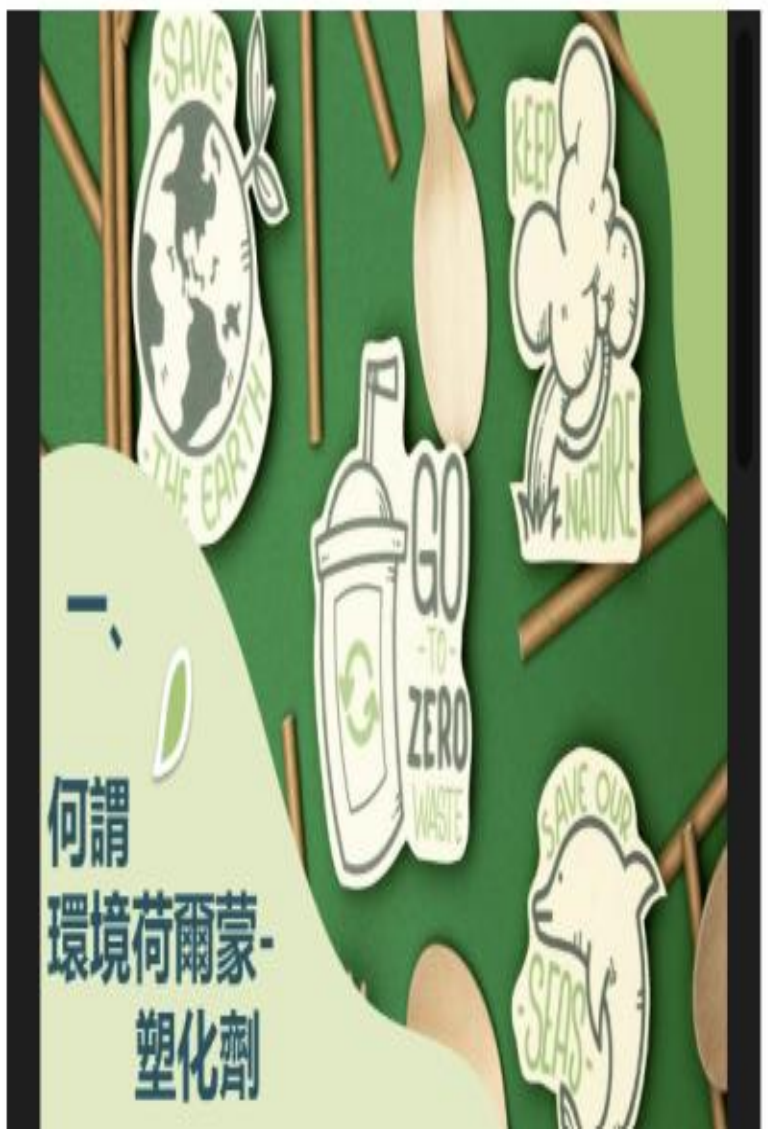

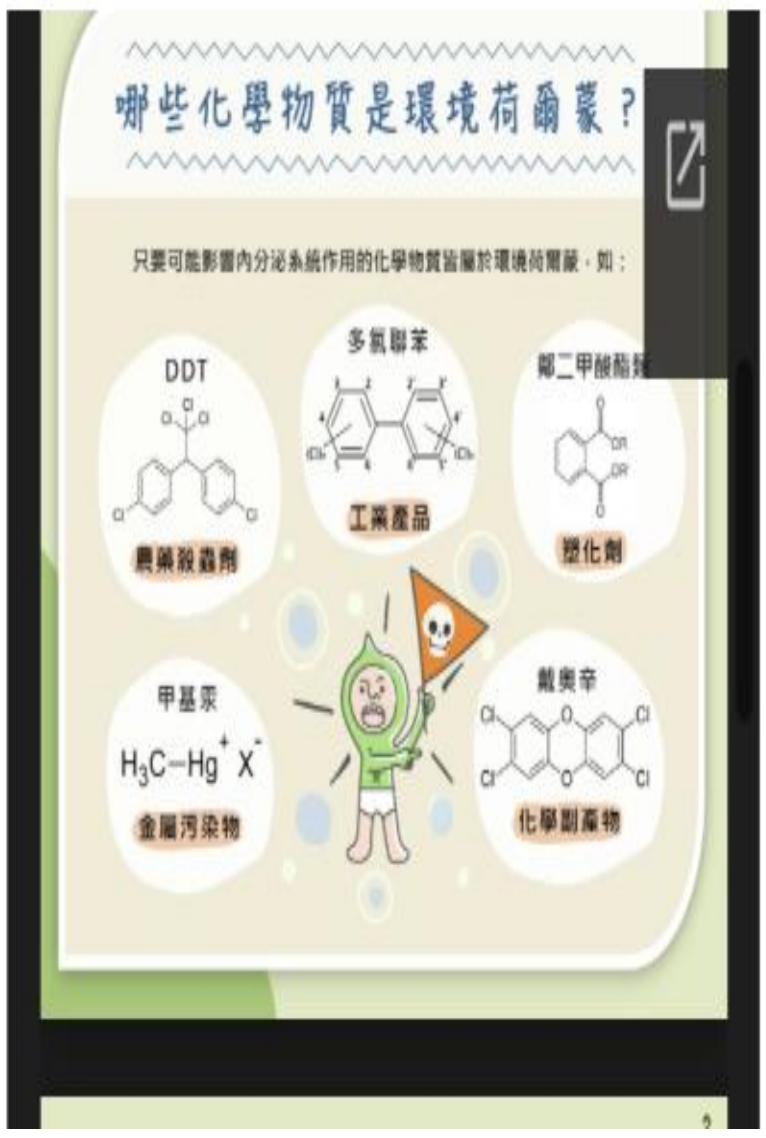

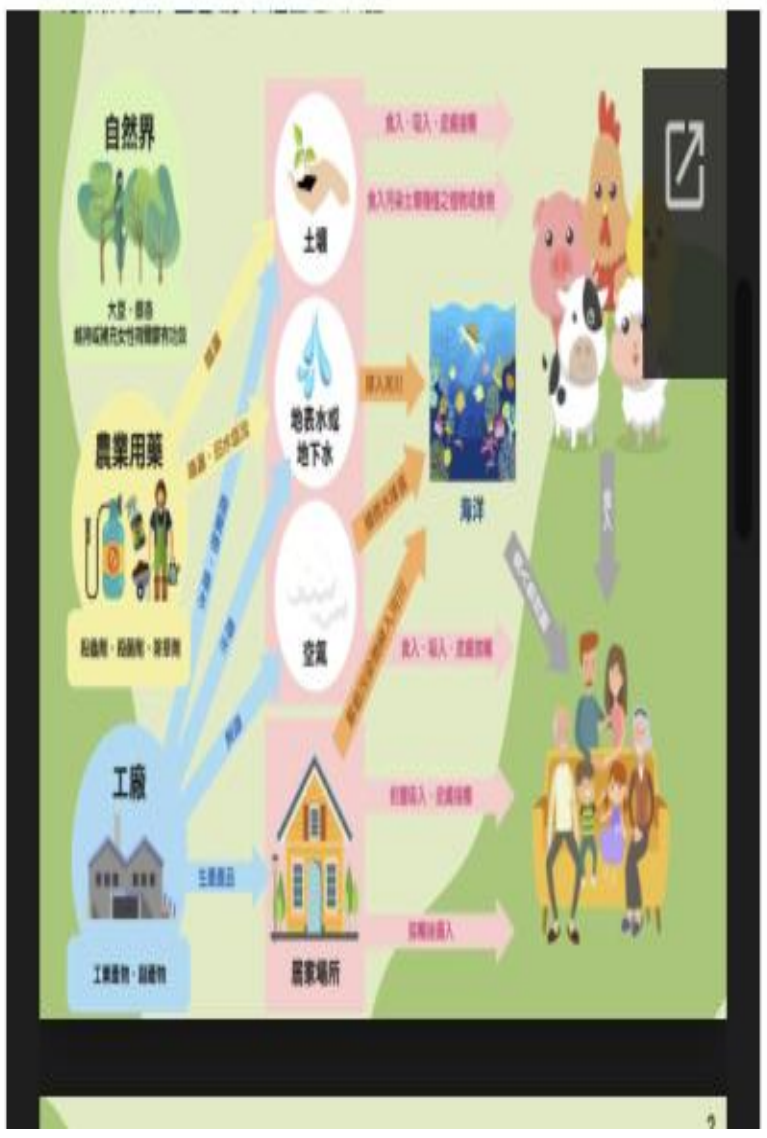

S2. Nursing health education modules

相關新聞

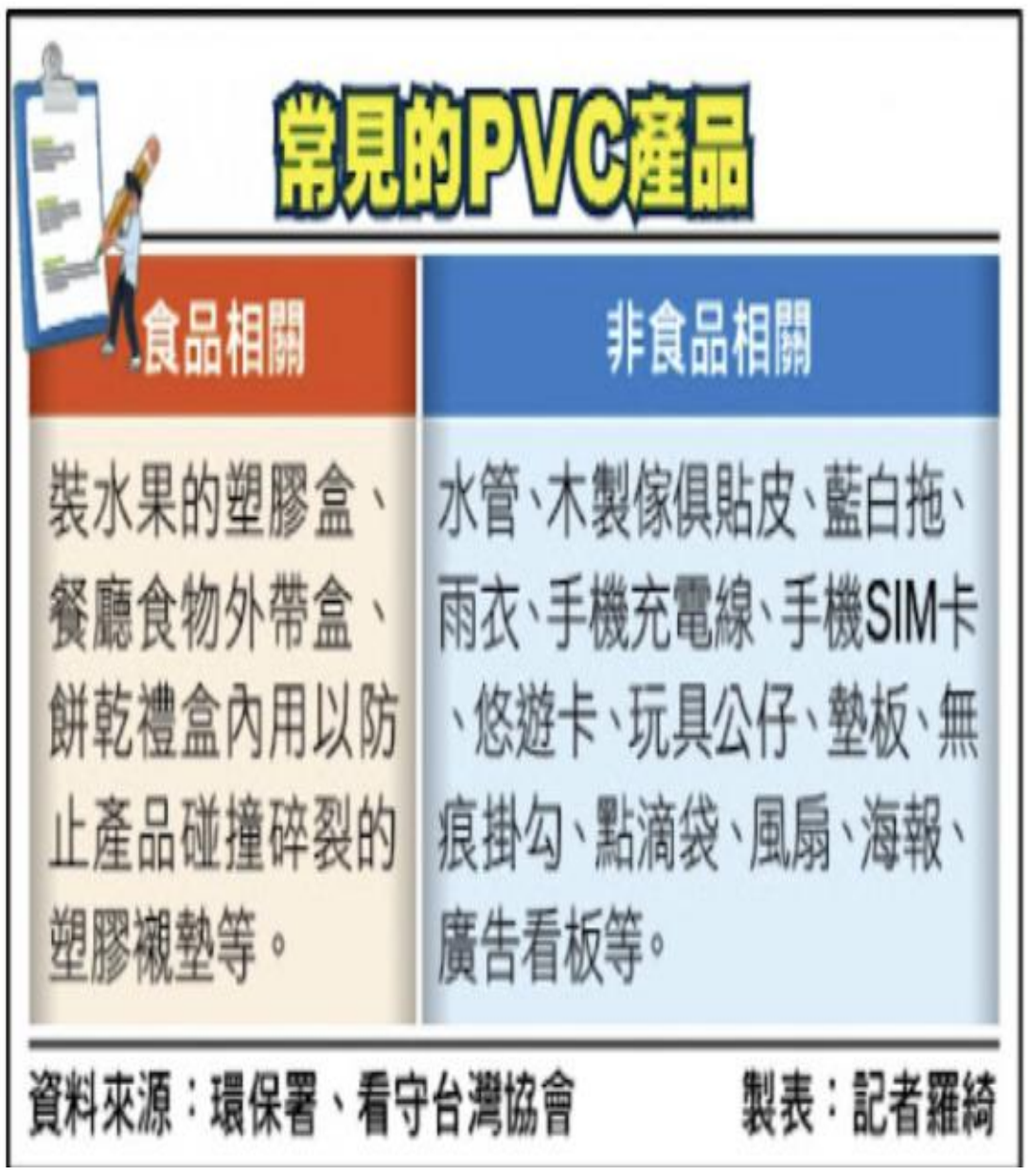

常見的PVC產品

食品相關	非食品相關
裝水果的塑膠盒、餐廳食物外帶盒、餅乾禮盒內用以防止產品碰撞碎裂的塑膠襯墊等。	水管、木製傢俱貼皮、藍白拖、雨衣、手機充電線、手機SIM卡、悠遊卡、玩具公仔、墊板、無痕掛勾、點滴袋、風扇、海報、廣告看板等。

資料來源：環保署、看守台灣協會　　製表：記者羅綺

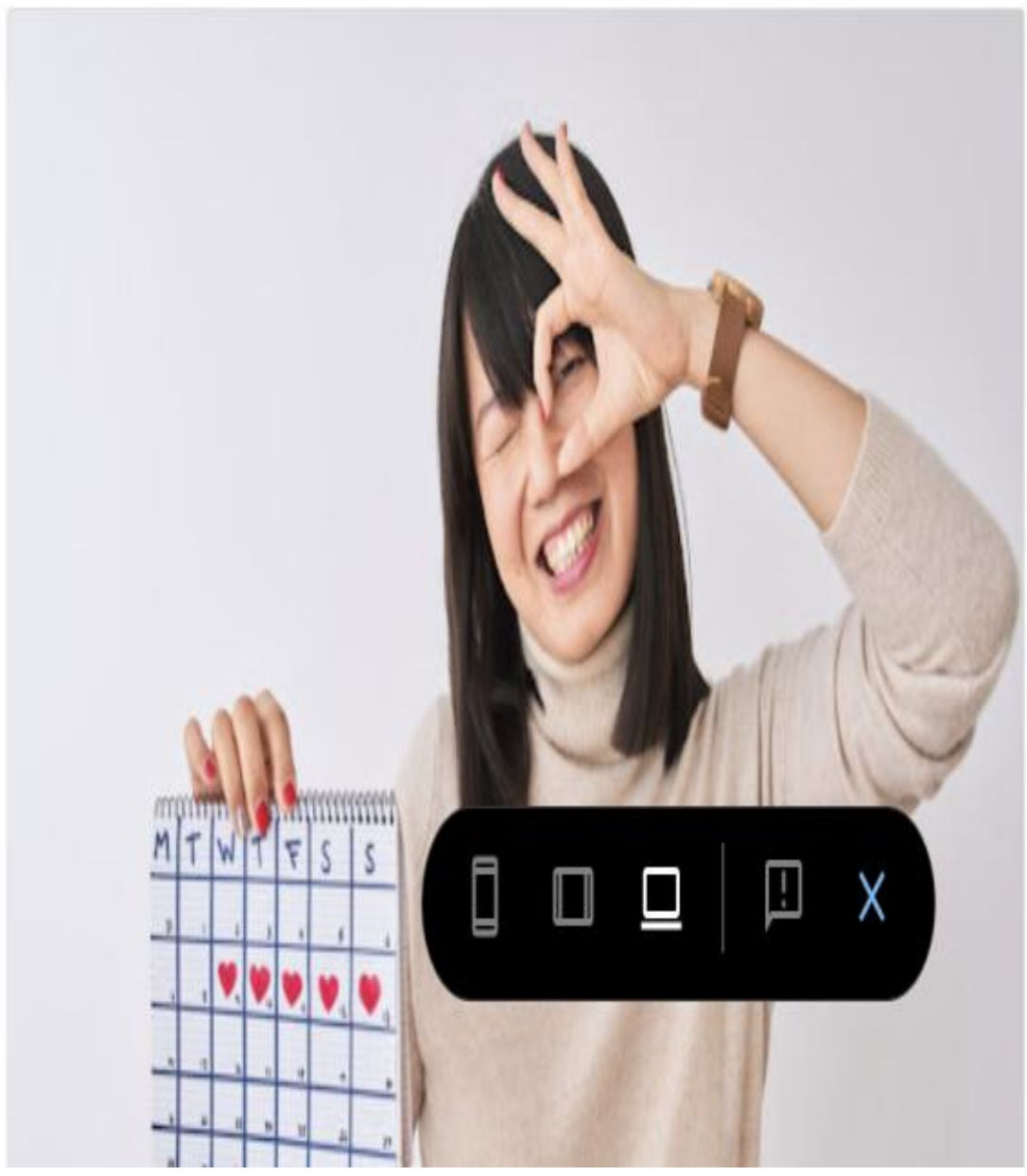

S3. Multimedia resources

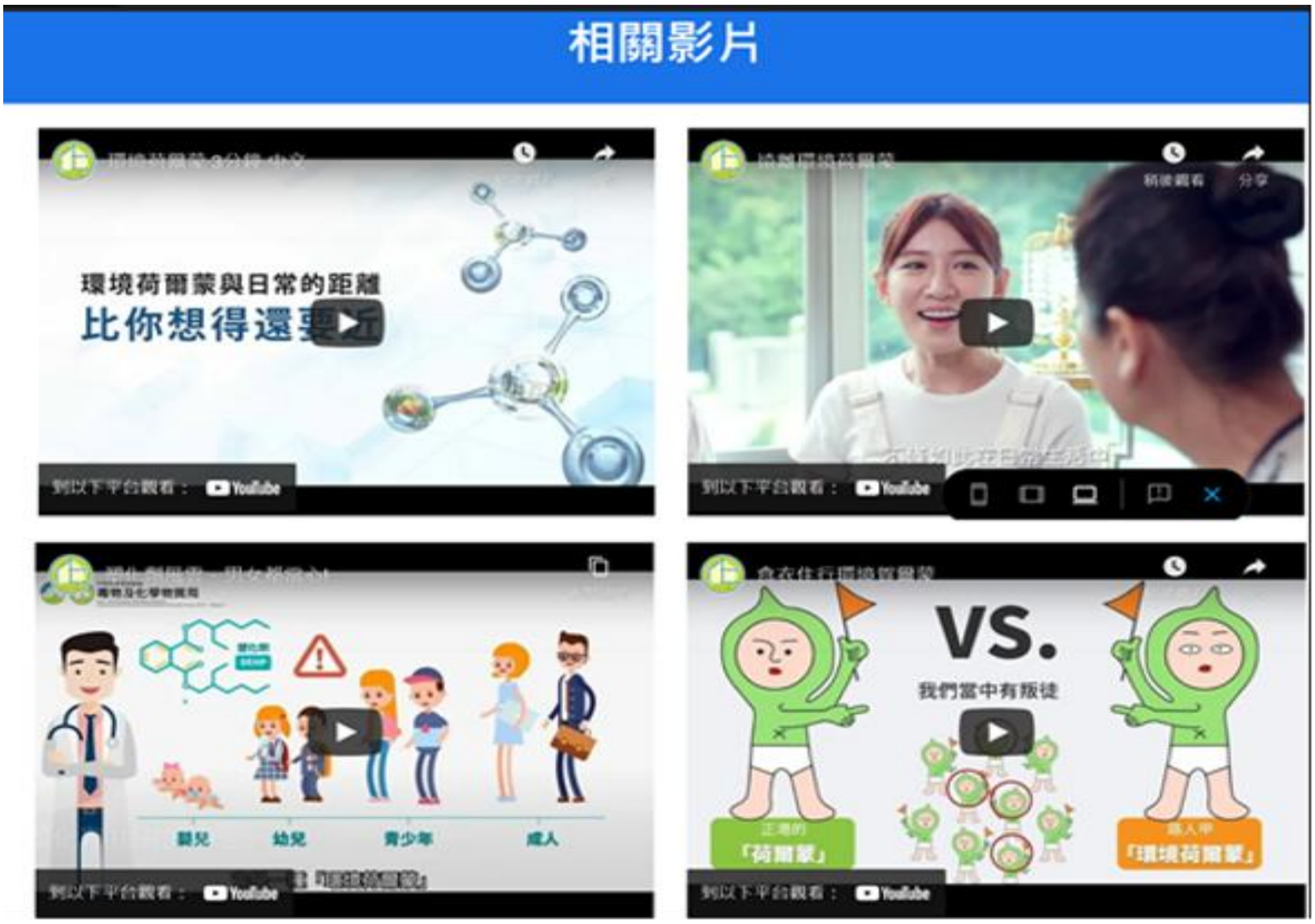

S4. Educational Video Links

生活小撇步

Q1.選擇回收編號為 1、2、4 或 5 的塑料，而不是 3、6 和 7。

A1.為什麼?

#3 和 #7 塑料可以釋出**環境荷爾蒙**，如雙酚 A (BPA)和塑化劑(鄰苯二甲酸鹽)，它們會干擾人體的天然荷爾蒙。一些鄰苯二甲酸鹽會影響生殖和發育，導致過敏和哮喘，並增加罹患癌症的風險。雙酚 A 是一種雌激素化學物質，與生育、兒童發育和生殖癌症的影響有關。請注意，標有 “不含 BPA” 的塑料和食品罐可能含有雙酚S (BPS)、雙酚F (BPF)或其他化學替代品。這些替代品尚未經過充分的安全測試。#6 塑料可以將與癌症有關的苯乙烯浸出到熱的或酸性的食物中，如蘇打水、咖啡或茶。

S5. Lifestyle Guidance

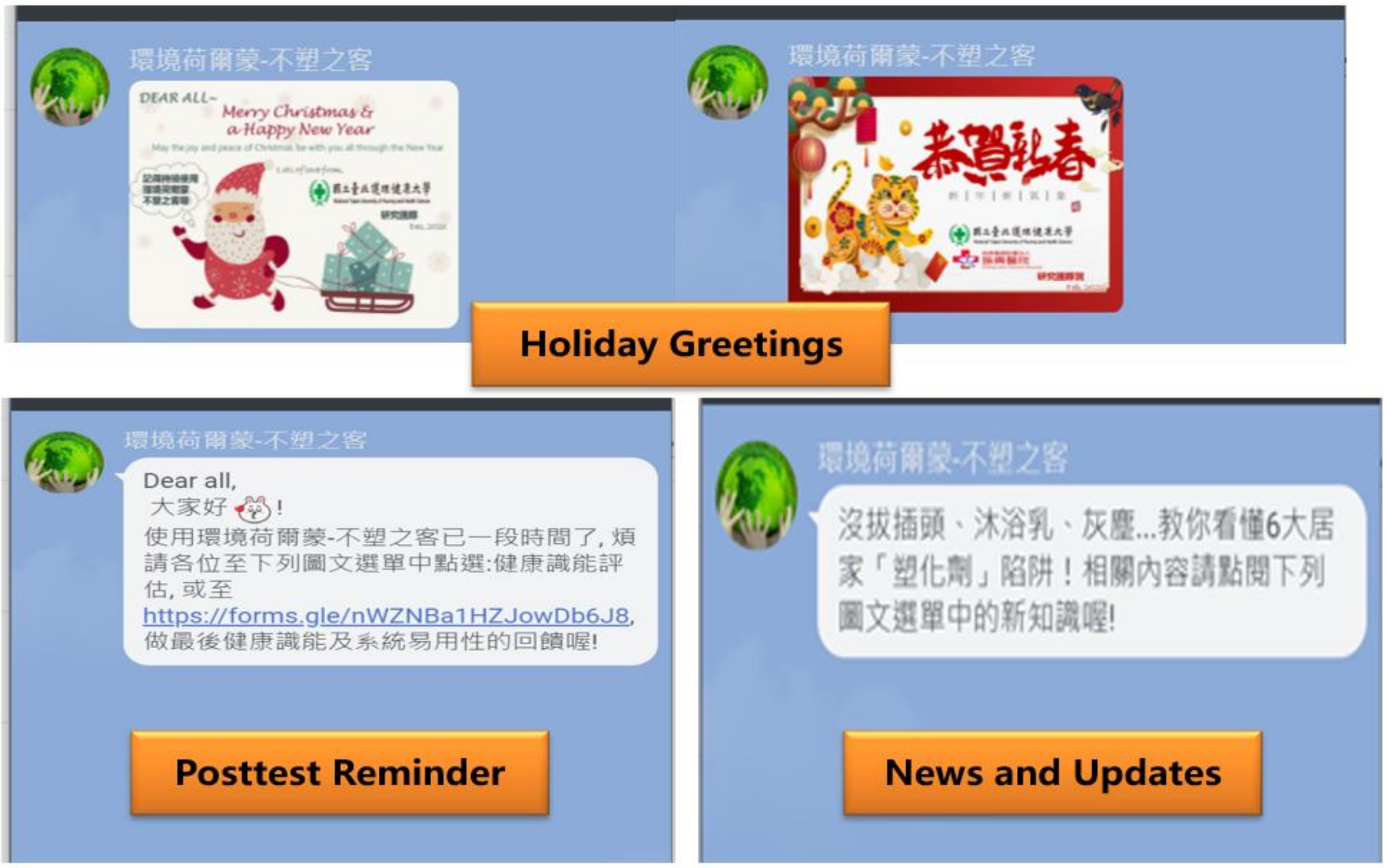

S6. Participant Engagement, Reminders, and Updates

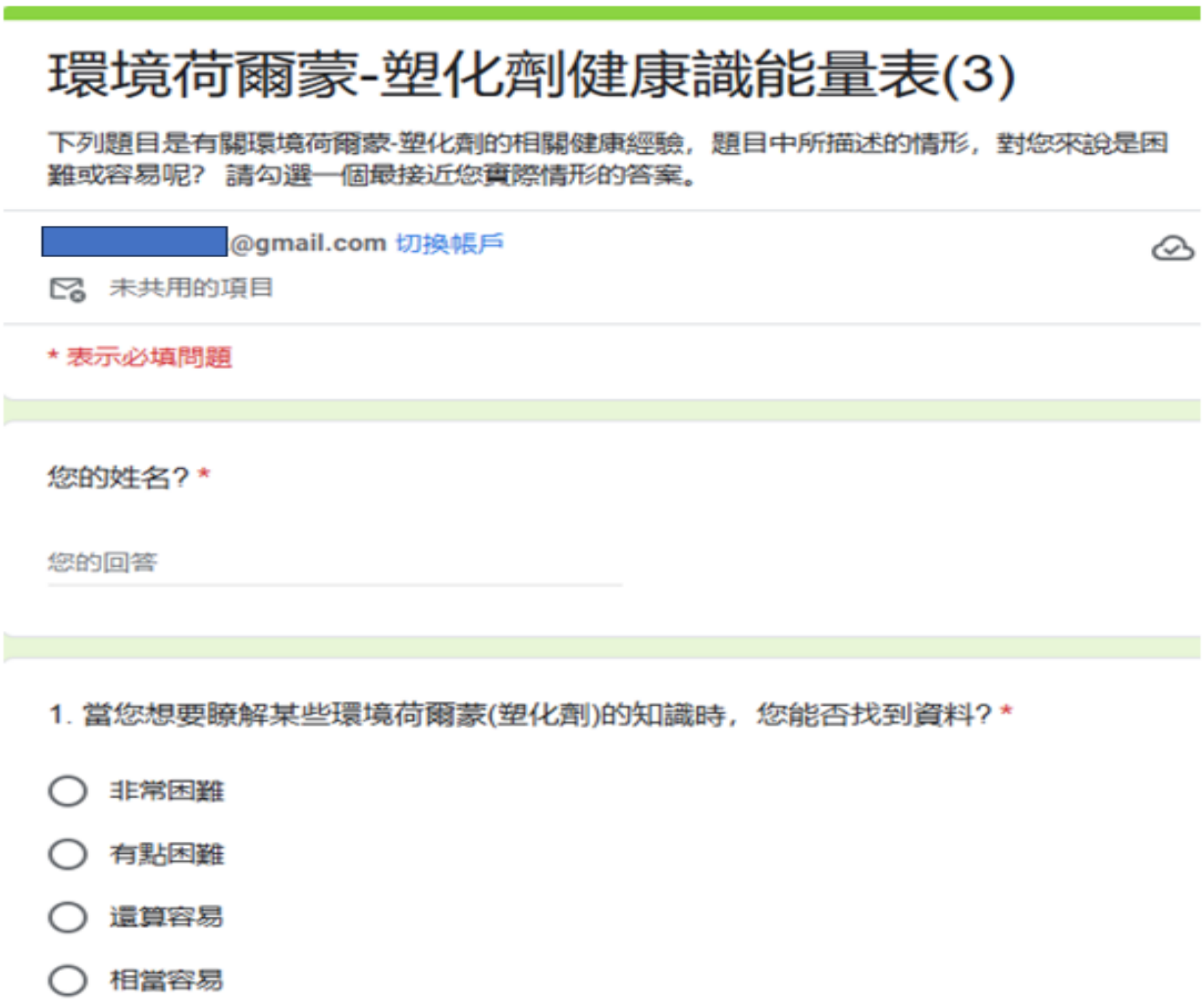

環境荷爾蒙-塑化劑健康識能量表(3)

下列題目是有關環境荷爾蒙-塑化劑的相關健康經驗，題目中所描述的情形，對您來說是困難或容易呢？ 請勾選一個最接近您實際情形的答案。

[redacted]@gmail.com 切換帳戶

未共用的項目

* 表示必填問題

您的姓名？ *

您的回答

1. 當您想要瞭解某些環境荷爾蒙(塑化劑)的知識時，您能否找到資料？ *

- ○ 非常困難
- ○ 有點困難
- ○ 還算容易
- ○ 相當容易

S7. Environmental Hormone–Phthalate Health Literacy Scale

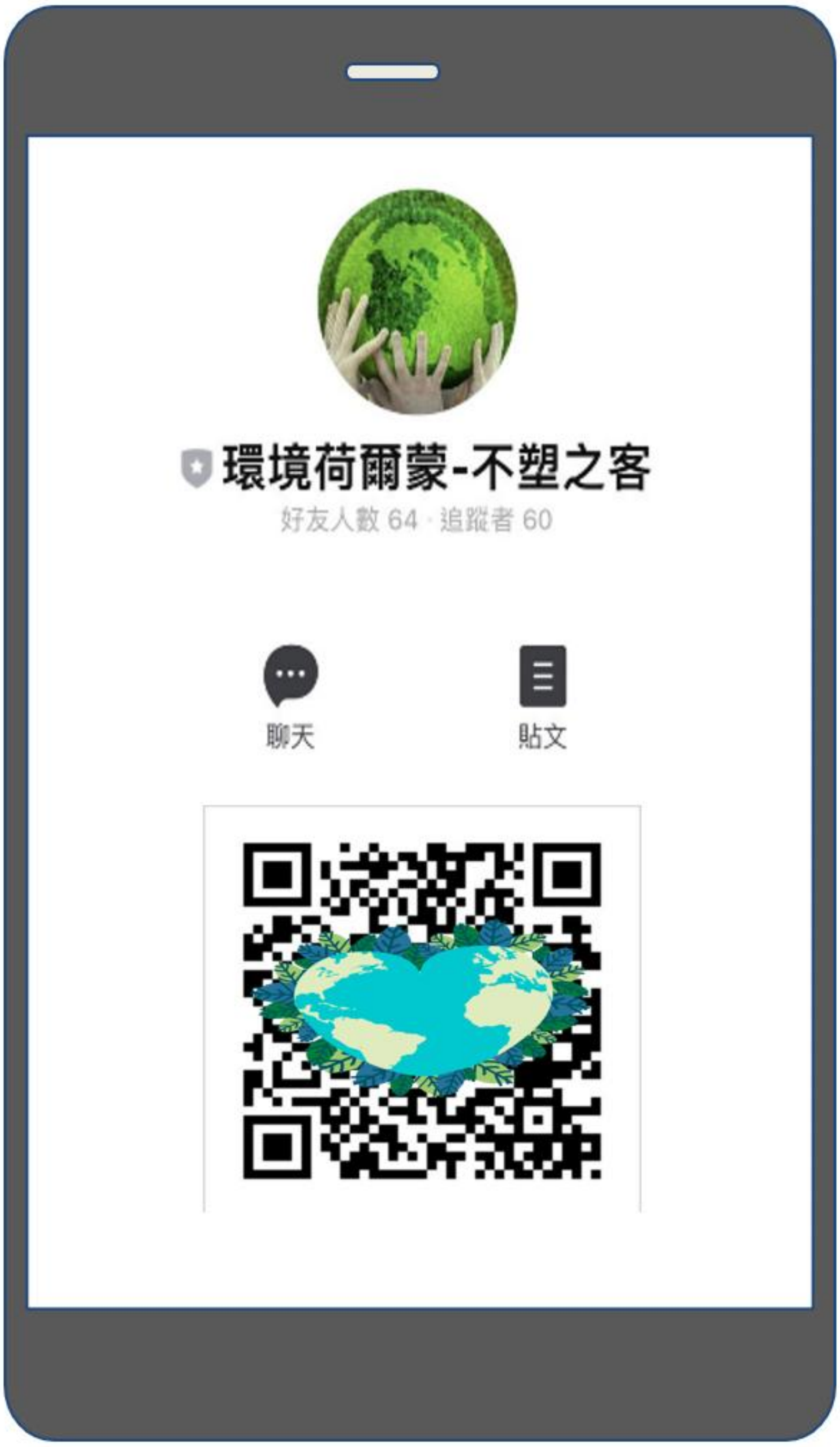

S8. Related External Links and QR-Code Access
